# The Candy Crush Sweet Tooth: How ‘Near-misses’ in Candy Crush Increase Frustration, and the Urge to Continue Gameplay

**DOI:** 10.1007/s10899-016-9633-7

**Published:** 2016-07-20

**Authors:** Chanel J. Larche, Natalia Musielak, Mike J. Dixon

**Affiliations:** University of Waterloo, 200 University Ave. West, Waterloo, ON N2L 3G1 Canada

**Keywords:** Near-misses, Frustration, Gaming, Arousal, Motivation, Gambling

## Abstract

Like many gambling games, the exceedingly popular and lucrative smartphone game “Candy Crush” features near-miss outcomes. In slot machines, a near-miss involves getting two of the needed three high-paying symbols on the pay-line (i.e., just missing the big win). In Candy Crush, the game signals when you just miss getting to the next level by one or two moves. Because near-misses in gambling games have consistently been shown to invigorate play despite being frustrating outcomes, the goal of the present study was to examine whether such near-misses trigger increases in player arousal, frustration and urge to continue play in Candy Crush. Sixty avid Candy Crush players were recruited to play the game for 30 min while having their Heart Rate, Skin Conductance Level, subjective arousal, frustration and urge to play recorded for three types of outcomes: wins (where they level up), losses (where they don’t come close to levelling up), and near-misses (where they just miss levelling up). Near-misses were more arousing than losses as indexed by increased heart rate and greater subjective arousal. Near-misses were also subjectively rated as the most frustrating of all outcomes. Most importantly, of any type of outcome, near-misses triggered the most substantial urge to continue play. These findings suggest that near-misses in Candy Crush play a role in player commitment to the game, and may contribute to players playing longer than intended.

## Introduction

Since the emergence of multimedia cellular phones in the mid-1990s, mobile phone gaming has claimed some degree of presence in our continuous, ‘on-the-go’ lifestyle. From the initial craze surrounding the game “Snake” on early Nokia devices, our mobile gaming habits have been transformed by the ever-expanding quality, sophistication and overall usage of smartphone technology. Of particular interest is the surging popularity of Candy Crush Saga—a free-to-play, candy themed puzzle game that has captivated at least 93 million daily active users in 2014, and generated $2.2 billion in profits (mostly from in-app purchases) in that same year (King Ltd 2015).

To play Candy Crush, players are allotted a fixed number of moves in which they can swap symbol positions with the goal of horizontally or vertically aligning three (or more) matching symbols. When matched symbols are aligned, points are awarded and the ‘captured’ matching symbols are removed from the game matrix (and replaced by other symbols). A single match constitutes one move. In each level of the game, the player must achieve a specific objective within a limited number of moves before the player can unlock the next level. The objectives can include bringing a certain number of ‘ingredient’ symbols to the bottom of the game matrix (in game play players are instructed to “Collect all 6 ingredients!”), or ‘freeing’ candy symbols encased in ‘gelatine’ or ‘jelly’ tiles (e.g. “Clear all the jellies!”). If the player meets the objective within the allotted number of moves they win, and move on to play the next level (colloquially known as “levelling up”). If they fail to meet the objective in the allotted number of moves they lose, and remain at the current level, which they must repeat if they wish to move on in the game.

Since games like Candy Crush are typically played on smartphones, they encompass a unique set of traits that distinguish them from console-type video games but intriguingly, bring them closer to the conceptual realm of slot machines. For example, like slot machines, smartphone games are easy to learn, and players are frequently reinforced as each successful move is accompanied by eye-catching animations of points being accrued as the aligned symbols are captured. Perhaps most importantly, play is continuous- there is always a next level to play (The Economist 2013). Although Candy Crush (like most phone games) lacks a direct gambling element in that no money is wagered on outcomes, money nevertheless can change hands. Players can, if they wish, purchase game currency that can be used to gain extra lives, extra moves or bonus accessories as a way to maximize their likelihood of winning and advancing in the game. Although less than 3 % of players end up making such transactions (Grubb 2014), the players who do, spend on average $23.42 per month on these micro-transactions (Grubb 2014).

The monetization of gaming through these micro-transactions blurs the dividing line separating regular video-gaming and gambling for money. In addition to the negative impact of excessive video-game play on overall social, physical and psychological well-being (Ferguson et al. 2011), some players can, and do spend more than they can afford on these games (Lloyd 2016).

### Structural Similarities Between Casual Games and Gambling Games

Several speculations comparing the structural similarities of Candy Crush and slot machines have been made in attempt to explain why Candy Crush has such an ‘addictive’ quality (see Smith 2014; Gardner 2014). Tellingly, Candy Crush players often specifically liken Candy Crush to slot machine play to convey its appeal. They highlight the enticing animations that accompany successful moves, and levelling up (Smith 2014). Moreover, the fact that a correct move is characterized by the alignment of matching candies parallels the alignment of matching symbols on the pay-line in slot machines. Furthermore, players attempting to gather (or capture) candies may allude to the indirect consumption of these forms of foods—a pleasurable experience which many of us are motivated to repeat (Lowe and Butryn 2007; Gardner 2014). Such game themes where food symbols are paired with reward are evident in many slot machines. In fact, in the United Kingdome, slot machines are colloquially referred to as ‘fruit’ machines (Griffiths 1993).

The parallels between slot machine play and Candy Crush involve not only rewarding events (winning spins, levelling up), but also frustrating events. Near-misses are outcomes that come close to, but fall just short of a win (Reid 1986). In traditional 3-reel slot machine games, a classic near-miss is represented by two high paying symbols matching up on the first two reels, and a 3rd matching symbol stopping right before or just after the pay-line (“7-7-X”). Thus, the player falls just short of the big win. In Candy Crush, the program specifically highlights attempts that fall just short of the goal of levelling up. For example, if the player needed only 2 moves to level up, but ran out of the allotted number of moves they would see the move counter drop to zero, followed by a message claiming “Out of moves! You only needed 2 more jellies”. In contrast, if the player was not close to levelling up, the move counter would simply drop to zero and the message would simply state “out of moves”. As such there is a clear attempt to highlight to the player those instances where players came close to, but fell just short of the goal of the game. We refer to these outcomes as Candy Crush near-misses.

Although no studies have investigated the ramifications of Candy Crush near-misses, one can make reasonable inferences based on near-misses in other scenarios. In slot machine games, near-miss outcomes encourage the urge to continue play despite the absence of reward (Côté et al. 2003; Kassinove and Schare 2001; Clark et al. 2009; Billieux et al. 2012). In general, the idea of falling just short of a big win appears to facilitate players wanting to continue with the game in the belief that practice makes better, or more spins will eventually lead to success (Kassinove and Schare 2001).

Because a near-miss reflects a thwarted goal, it tends to provoke a negative emotional experience. While players rate slot machine wins as being pleasant, they rate near-misses as being unpleasant and more aversive than regular losses (Clark et al. 2009; Chase and Clark 2010). One means of capturing the rewarding property of wins and the aversive property of near-misses during actual play is by measuring a combination of Post-reinforcement Pauses (PRPs) and Skin Conductance Responses (SCRs). Post-reinforcement pauses are typically defined as the time it takes to initiate a new response after a specified reinforcement (Felton and Lyon 1966). In slot machine play, PRPs are operationalized as the time interval between the delivery of an outcome (e.g. win, loss, or near-miss) and the initiation of the next spin (Dixon et al. 2013; Dixon and Schreiber 2004; Delfabbro and Winefield 1999). After having participants play a slot machine, Dixon et al. (2013) found relatively long PRPs for winning outcomes compared to near-misses (and other standard losses). Players’ faster initiation of the next spin following a near-miss outcome was seen as an attempt to escape the unpleasantness of just missing the win (Dixon et al. 2013). Research measuring arousal (quantified by SCRs) complement this interpretation (Lobbestael et al. 2008; Civai et al. 2010). During slot machine play, wins trigger significantly larger arousal responses than losses, presumably due to their exciting properties. Near-misses, however also trigger large skin conductance responses than regular losses—a finding Dixon et al. (2011, 2013, 2015) attributed to their frustrating properties. In sum the combination of long PRPs and large SCRs was viewed as a signature of reward-induced arousal, whereas the combination of large SCRs but small PRPs was seen as a hallmark of frustration. Based on the slot machine literature, it is reasonable to surmise that near-misses in Candy Crush (just failing to level up by one, two or three moves) might induce similar frustration that could be operationalized by the combination of large elevations in skin conductance and short PRPs.

Near-misses influence players in different forms of gambling. For example, a recent study by Stange et al. (in press) investigated near-misses in scratch card play. Players uncovered a series of symbols hoping to find three matching symbols within a 3 × 2 symbol matrix. They compared losing outcomes (no matching symbols), winning outcomes (three $5 symbols leading to a small win) and near-miss outcomes (where only two of three “jackpot” symbols were uncovered and players “just missed” winning a large prize). In such a game, the outcomes are only known once the last symbol in a matrix is revealed. Stange et al. (in press) showed that during near-misses (compared to regular losses), as players successively revealed a first, then a second jackpot symbol their skin conductance levels (SCLs) increased presumably due to increases in arousal in anticipation of the big win. Elevations in Heart Rate (HR) also took place during near-misses as the first and second symbol were uncovered. They also found that subjective frustration ensued when players uncovered the last symbol and their hopes were dashed. We surmise that this anticipatory build up as players get closer and closer to their goal and the frustration encountered when they “just miss” achieving their goal may occur not only during scratch card play but also during Candy Crush gameplay. As players make more and more moves, they accrue points and get closer and closer to levelling up. When they run out of moves it is reasonable to assume that frustration will ensue.

To summarize, most current research on near-misses pertains to studies of gambling, limiting their application to the smartphone gaming context. Here we will examine how winning (levelling up), losing, and just failing to win (a near-miss) in Candy Crush affects players’ levels of physiological arousal (as indexed by HR and SCL), emotional reactions (as indexed by subjective ratings) and reward responses (indexed by PRPs). We hypothesize that near-miss outcomes will produce greater physiological arousal (higher HR and SCL) than full loss outcomes during the game. Here, following Stange et al. (in press), we will measure SCL changes that occur during the game as players get closer and closer to levelling up. We expect near-misses to trigger similar SCL changes to actual wins since the anticipatory build up period prior to winning or proximally winning should be comparable. Based on previous findings by Dixon et al. (2013), we hypothesize that players will produce longer PRPs following wins than either regular losses or near-misses. If indeed we see high arousal but small PRPs for near-misses (the aforementioned signature of frustration), we should also see greater subjective ratings of arousal and frustration for near-misses than for losses. Finally, consistent with gambling studies where near-misses trigger the urge to continue gambling, we predict that subjective ratings of urge to continue play will be stronger for near-misses compared to regular losses.

## Methods

### Participants

A total of 60 Candy Crush Saga players were recruited to participate from two pools of students at the University of Waterloo. The first pool consisted of students participating in studies advertised for extra credit in a psychology course of their choosing. Students in this pool were recruited through the University of Waterloo’s SONA system—a website that manages student participation in Psychology studies. Out of the 323 students from this pool who were eligible to participate, 39 participated. The second pool consisted of students who voluntarily enrolled in a pool to participate in experiments for financial remuneration. These students submitted their contact information to the department of Psychology to be included in a database accessed only by authorized researchers. A total of 141 students from this pool were contacted. Out of this number, 22 participants responded, and 21 participated. Students recruited from this pool were compensated $10 for their time.

Students from both pools were first asked to complete a pre-screen survey to ensure: (1) students reached at least level 70 in the Candy Crush Saga, and (2) students had played the Candy Crush Saga within the last 12 months. Assigning a cut off level of 70 in the Candy Crush Saga ensured that players were adequately experienced players.

The final results of the study are based on 57 students (48 female) between the ages of 18-24 (M = 21, SD = 1.43). Participants were excluded if they did not meet the aforementioned criteria or if there were issues in data collection (e.g. technical issues, etc.). Participants on average had achieved level 287 (ranging from 70 to 930). In terms of playing frequency, 23.8 % of players reported playing Candy Crush on a daily basis, 65.7 % reported that they played the game at least twice a week, and 10.5 % reporting that they rarely play.

The current study’s protocol was reviewed and approved by the University of Waterloo Research Ethics Committee. All participants were provided sufficient information about the study prior to participating, and were advised that they could withdraw at any point in the study without penalty.

### Apparatus

#### Candy Crush Saga Game

**Fig. 1:**
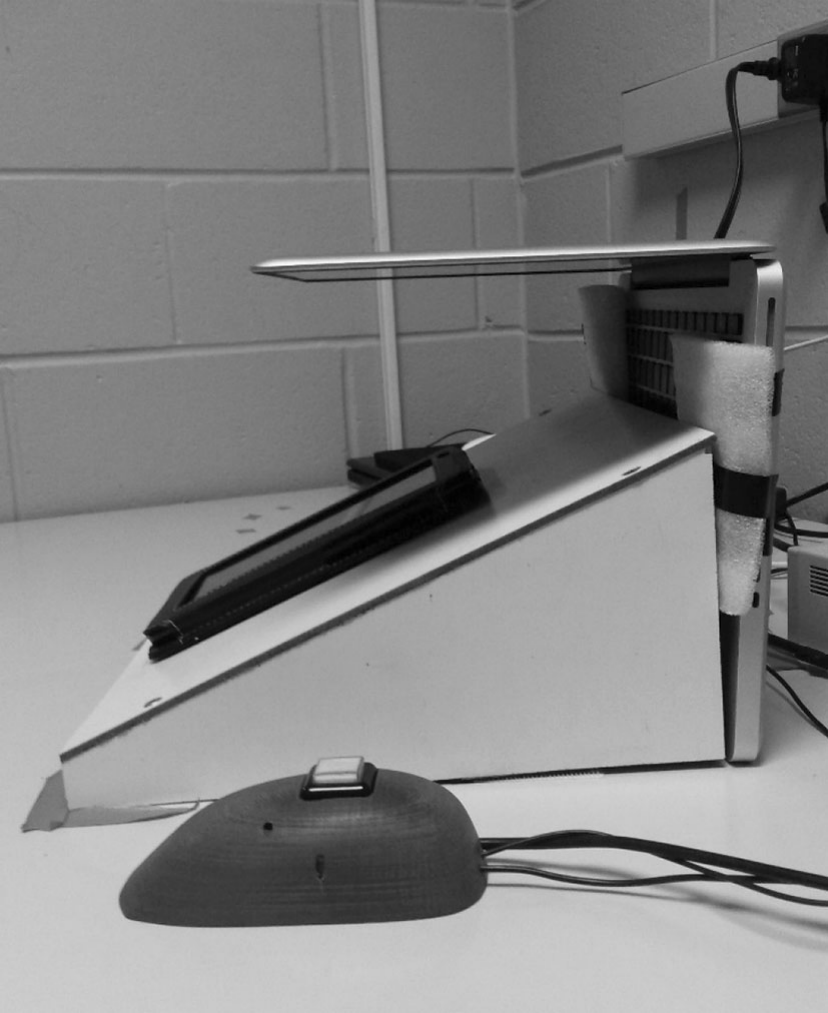
Specialized platform used to hold the Lenovo tablet upright. A MacBook pro camera was used to record the players’ game screen in order to time-lock game events (e.g. wins, losses and near-misses). Beside the platform is a *button* *box* used to measure PRPs

Participants played a real, complete version of Candy Crush on an Android tablet device. A built-in video camera on a MacBook Pro laptop was used to capture the tablet screen as participants played the game. The tablet rested on a tilted platform aligned to the computer’s camera. The video was used to record the outcomes (pictured in Fig. 1) that were delivered during game play and mark the precise time of their delivery for data analysis.

#### Baseline Task

Participants traced their finger following a moving dot on a rotary pursuit wheel presented on the screen of the tablet device. Participants did this 3 times, once at the beginning, midway, and at the end of 30-min gameplay for a period of 120 s each.

#### Heart Rate

**Fig. 2:**
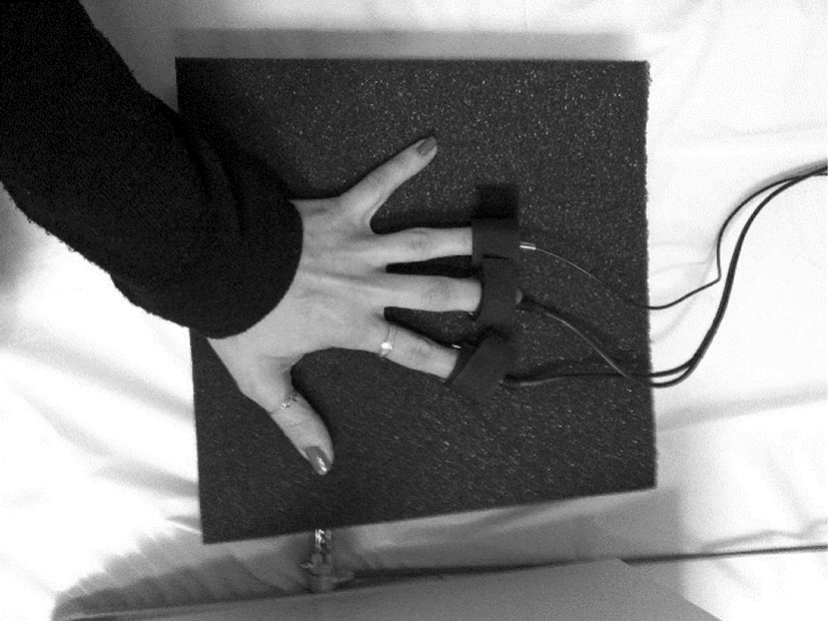
Pulse Transducer and metallic Skin Conductance electrodes. Participants rested their hand on a foam block during play

Heart rate was recorded using an ADinstruments TN1012/ST pulse transducer attached to the participant’s ring finger (pictured in Fig. 2). The pulse transducer was fed into a ML866 Powerlab (model 4/30), which amplified the signal and provided a digital recording of participants’ physiological responses.

#### Skin Conductance

Skin Conductance Level (SCL) was recorded using two small metallic plates (ADinstruments MLT116F electrodes) attached to the participant’s index and middle finger (see Fig. 2). The electrodes were also fed into the same ML866 Powerlab (model 4/30).

#### Post-reinforcement Pause

**Fig. 3:**
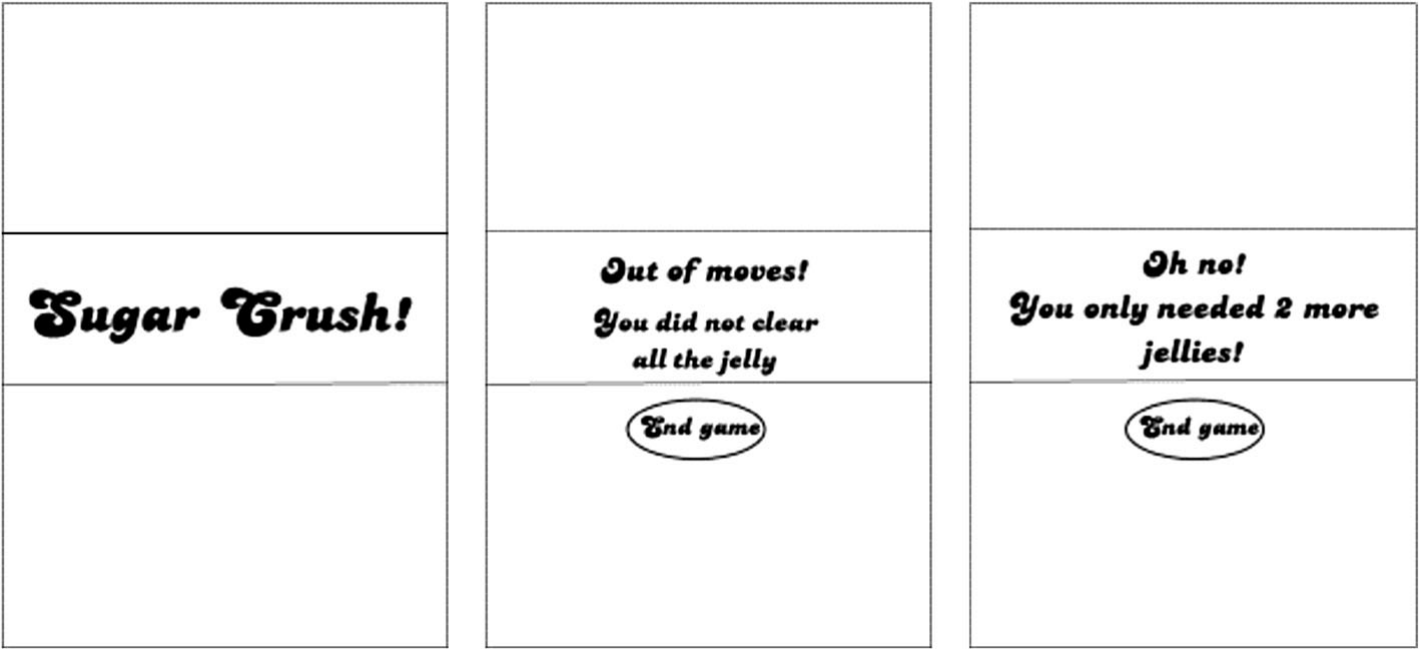
Outcome delivery messages in Candy Crush. These messages are what players would see for wins (*left*), losses (*center*), and near-misses (*right*)

Post-reinforcement pauses (PRPs) were defined as the delay between an outcome delivery in one game and the initiation of the next game, measured in seconds. In Candy Crush, a message at the end of each game appears. The messages associated with the three different outcomes are shown in Fig. 3. Players were instructed to press a button on a button box adjacent to the tablet when they were ready to answer a set of subjective surveys and play the next game. The post reinforcement pause for any given outcome was the total time delay between the appearance of the outcome message and when they pressed this button.

### Materials

#### Pre-test Questionnaire

Prior to commencing the study, participants were asked to complete a brief questionnaire (using the Qualtrics survey system) composed of demographic information (age, gender), as well as their experience with the smartphone game (current Candy Crush level, playing frequency, and an estimate of the amount of time they allocated to the game).

#### Subjective Rating of Arousal

**Fig. 4:**
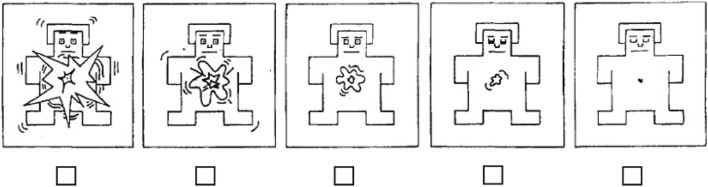
Self-assessment Manikin (SAM) used to rate subjective arousal following each outcome type (i.e., losses, wins and near-misses)

The Self-Assessment Manikin (SAM; Lang 1985) measure is a non-verbal, self-report tool used to measure one’s immediate experience of arousal. A single-item arousal scale was employed, with each item pictorially represented by a manikin displaying different degrees of arousal intensity (Fig. 4). Participants were asked to indicate their level of arousal by placing an ‘x’ under the corresponding picture immediately following the delivery of each game outcome that they experienced during the study.

#### Subjective Rating of Frustration

Subjective frustration was measured by having participants evaluate how much they agree/disagree with the statement “I feel frustrated” on a 7-point Likert scale after each game outcome was delivered. The scale ranged from 1 to 7, with 1 representing ‘*Strongly* *Disagree*’ and 7 representing ‘*Strongly* *Agree*’.

#### Subjective Ratings of Gaming Urge

Two items derived from The Gambling Urge Scale (GUS) measured urge to continue playing the game following each outcome experienced. These two items included “All I want to do is keep playing” and “I want to play so badly that I can almost feel it”, and were reworded to exclude gambling terminology. Participants were asked to rate their desire to continue playing using a 7-point Likert scale, with 1 indicating ‘*Strongly* *Disagree*’ and 7 indicating ‘*Strongly* *Agree*’. Both items of urge were summed (in accordance to scoring guidelines of the regular GUS), and averaged (i.e., all summed GUS scores for wins were averaged).

### Design

The present study consisted of gameplay epochs and baseline epochs. During gameplay epochs each participant would play up to eight games of Candy Crush. During the eight games, players were expected to encounter all three possible outcome types, (wins, losses and near-misses). Players were instructed to play a ninth game if they did not experience all three outcome types within the initial eight games. Each outcome was demarcated by a specific message at a game’s end: a ‘sugar crush’ demarcated a win, an ‘out of moves’ message demarcated a regular loss, and a message unambiguously specifying how close the player was to a win (e.g. “You only needed two more jellies!”) demarcated a near-miss (refer to Fig. 3). An a priori decision was made to only consider as near-misses those outcomes whose proximity message indicated that they were three moves away or less.

Baseline epochs occurred: just prior to game play, after the player had completed four games, and after eight games had been completed.

### Procedure

Participants were invited into the testing room and were first asked to complete a consent form, and the pre-test questionnaire. Following the completion of the questionnaire, the skin conductance electrodes and the pulse transducer were attached to their left hand and placed on a foam block.

Once the electrodes were attached, the experimenter provided the participant with verbal instructions for game play and baseline epochs. All participants started with an acclimatization period. They were given 3 min to play two easy practice games (level six and level seven in the Candy Crush Saga). This allowed the participant to adjust to playing the game with the electrodes attached to their left hand. Next was the first baseline task where participants completed a digitized rotary pursuit task on the tablet. They simply traced their finger over a dot moving in a circle. Following the baseline task, participants then played four games on the tablet device. To maximize the probability that participants would experience wins, losses and near-misses during gameplay, the researcher preselected game levels set at 15 levels below his or her personal best. Each of the four games took approximately 5 min to complete. Before initiating the gameplay epoch, participants were shown a button box placed beside the tablet (refer to Fig. 1). They were told to press the button when they wished to start gameplay, and to press the button once again when the game was over. Once players indicated that the game had ended (via a button press), they were administered the self-report items assessing subjective arousal, frustration, and urge to continue playing. This questionnaire was administered following each game. After answering questions related to the fourth game, a second baseline session (rotor pursuit task) was administered followed by four more games, followed by a final baseline epoch. The conditional ninth game followed the 3^rd^ baseline epoch.

## Results

Out of the 60 participants recruited, only 56 had valid data for all measures. Three participants were excluded because they did not experience all three game outcomes and one had a technical issue with the recording software. Participants ranged in their frequency of play from those who played very seldom to those who played multiple times per day. Most participants reported playing between 20 and 30 min in a game session. Frequency of play and reported session lengths are shown in Tables 1 and 2.

**Table 1:** Baseline playing frequencies on a weekly basis

Weekly playing frequency (*N* = *56*)	# Responses
Daily, multiple times a day	12
7 or more times a week	7
5–6 times a week	5
3–4 times a week	5
1–2 times a week	21
Rarely, almost never	6

**Table 2:** Reported session length

Session length (*N* = *56*)	# Responses
3–4 h	0
1–2 h	4
40–50 min	10
20–30 min	35
0–10 min	7

### Data Reduction and Analysis Strategy

All measures were subject to outlier rejection analyses. Data points more than 3 standard deviations away from the mean were considered outliers. Heart rate was measured in Beats per Minute (BPM). To circumvent the fact that games could be of different temporal lengths we analyzed BPM and SCLs only for the last 30 s of each game (ending with the posting of one of the outcome delivery messages in Fig. 3), and the last 30 s of the baseline periods. Changes in SCL were measured by calculating the slope of SCLs over this 30-s epoch. For all measures, outcomes of the same type were averaged. For instance, if the player experienced four losses, two wins and two near misses, the data for each measure would be reduced to three numbers (e.g. there would be three arousal ratings comprised of the average for losses, the average for wins and the average near-misses). For data analyses involving baselines (HR and SCLs), there were four data points per participant- three data points related to game outcomes (average of wins, average of losses, average of near-misses) and one data point reflecting the average of the baselines. For HR, SCLs and PRPs, we conducted repeated measures analyses of variance (ANOVA) involving all outcomes followed by Fisher’s Least Significant Difference (LSD) Comparisons. In instances where there were violations of sphericity, Greenhouse-Geisser corrections were applied.

For the subjective data we employ planned contrasts between near-misses and losses, wins and losses, and wins and near-misses for all of the subjective measures. Note main effects in an analyses of variance would be underpowered since for many measures no difference was predicted between two of the three means. For example, similarly high arousal should occur for wins and near-misses.

#### Physiological Measures

**Fig. 5:**
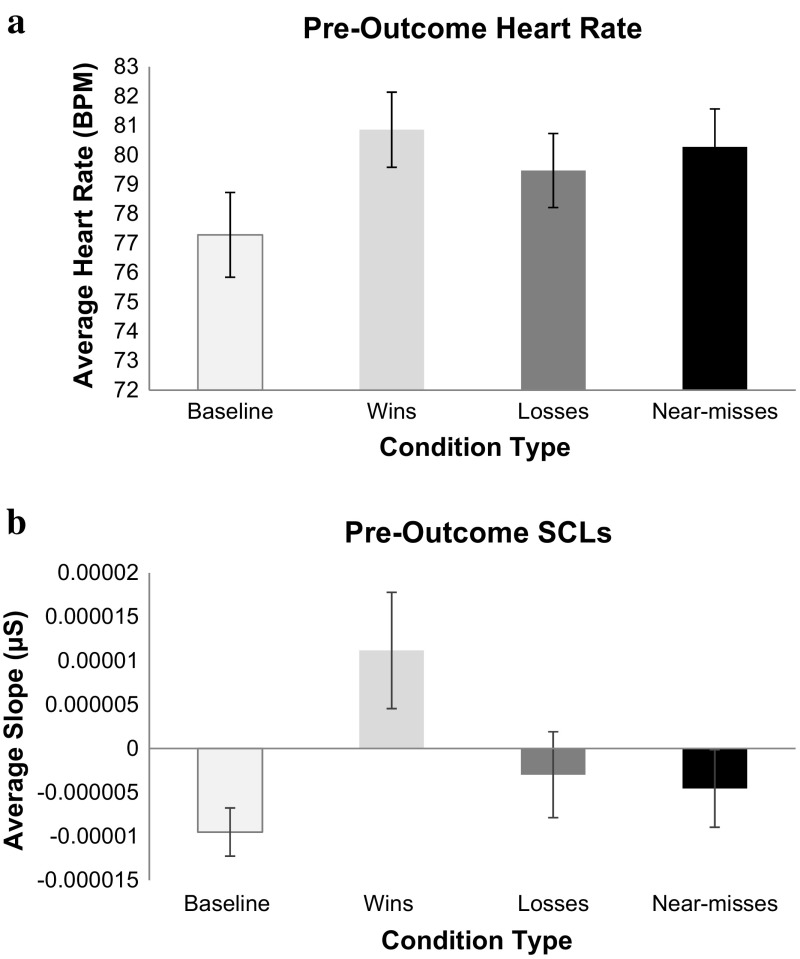
**a** Average BPM sampled 30 s prior to the end of each condition type. *Error* *bars* ± 1 SE. **b** Average SCL sampled 30 s prior to the end of each condition type. *Error* *bars* ± 1 SE

As shown in Fig. 5, the baseline condition was associated with the lowest heart rate. During game play, the 30 s leading up to either a win or a near-miss appeared to trigger relatively high heart rates, with losses triggering lower heart rates. A repeated measures ANOVA with a Greenhouse-Geisser correction revealed a significant main effect of condition, *F*(2.427, 133.50) = 18.75, *p* < .001, *η* ^2^ = .25. Fisher’s LSD post hoc tests showed that baseline heart rate was slower than any of the game play outcomes (*p* < .001 for all values). Near-misses triggered significantly higher heart rates than regular losses (*p* = .05) but the average HR for wins did not statistically differ from near-misses (*p* = .30). Wins had higher heart rates than regular losses (*p* = .03).

The changes in skin conductance levels (SCLs) over the final 30 s leading up to a win, loss or near-miss (or the last 30 s of the baseline period) are also shown in Fig. 5. This figure shows a general reduction in SCLs over time during the baseline period, an increase in SCLs over time for wins, and little change for losses and near-misses. A repeated measures ANOVA with a Greenhouse-Geisser correction indicated a significant main effect of SCL change by condition type, *F*(2.11,107.79) = 3.11, *p* = .04, *η* ^2^ = .06. Fischer’s LSD post hoc comparisons showed significantly larger slope increases for wins compared to near-misses (*p* = .02), but not losses (*p* = .13). Losses and near-misses were not statistically different (*p* = .83). The baseline epoch had significantly lower slopes than wins (*p* = .01) but not near-misses (*p* = .34) or losses (*p* = .21).

#### Post-reinforcement Pauses

Near-misses (*M* = 1.85, *SD* = .89), and losses (*M* = 1.92, *SD* = 1.03) had short PRPs compared to wins (*M* = 12.05, *SD* = 8.85). Repeated measures analyses with a Greenhouse Geisser correction indicated that there was a significant main effect of outcome type, *F*(1.01, 50.76) = 71.29, *p* < .001, *η* ^2^ = .58. Post-hoc comparisons indicated that PRP lengths for near-misses did not statistically differ from PRPs for losses (*p* = .60). However, PRPs for wins were statistically longer than PRPs for losses (*p* < .001) and near-misses (*p* < .001).

#### Subjective Measures

**Fig. 6:**
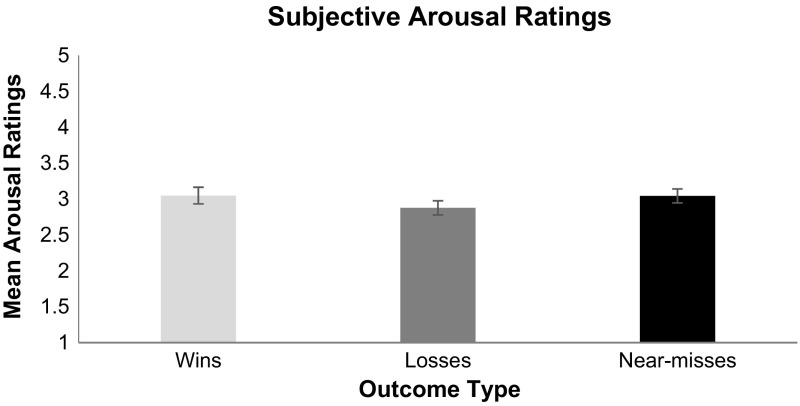
Subjective ratings of arousal for each outcome type on a scale from 1 (least aroused) and 5 (most aroused). *Error* *bars* are ± 1 SE

Average arousal ratings are shown in Fig. 6. The planned comparison between arousal ratings for near-misses and losses revealed that near-misses were more arousing outcomes than regular losses, *t*(56) = 2.16, *SE* = .077, *p* = .03. By contrast, the planned comparison of arousal ratings between wins and losses was not significant, *t*(56) = −1.37, *SE* = .124, *p* = .17, nor was the planned comparison between arousal ratings for wins and near-misses, *t*(56) = .05, *SE* = .05, *p* = .95.

**Fig. 7:**
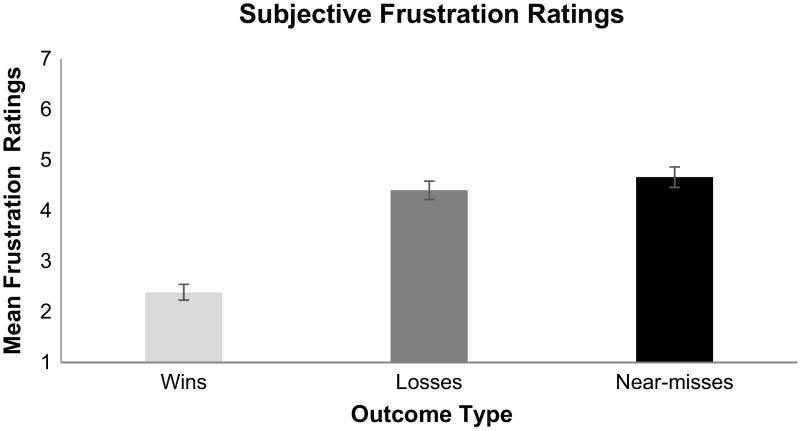
Subjective ratings of frustration for each outcome type on a scale from 1 (no frustration) to 7 (extremely frustrated). *Error* *bars* are ± 1 SE

For frustration (shown in Fig. 7), near-misses had the highest frustration ratings, followed by losses, and wins. The planned comparison between frustration ratings for near-misses and losses indicated that near-misses were significantly more frustrating than losses, *t*(56) = 2.01, *SE* = .12, *p* = .04. Expectedly, frustration following wins was statistically lower than losses, *t*(56) = 10.50, *SE* = .19, *p* < .001, and statistically lower than near-misses, *t*(56) = −10.41, *SE* = .21, *p* < .001.

**Fig. 8:**
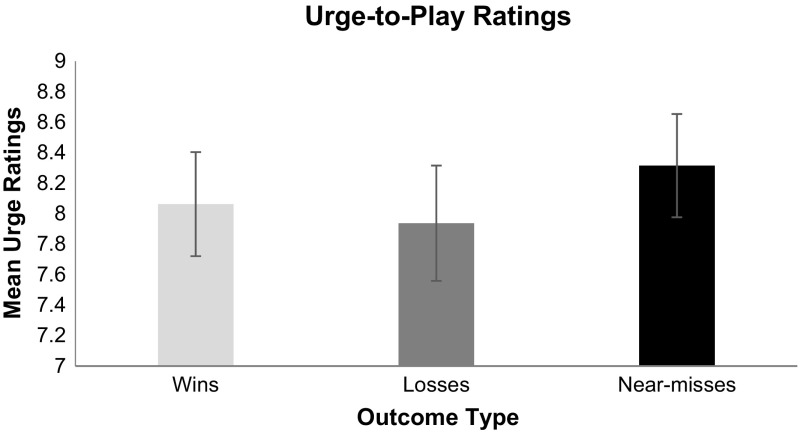
Urge to play for each outcome type. Two items measured urge on a scale from 1 (least urge) to 7 (most urge), and were scored by summing the two obtained values. *Error* *bars* ± 1 SE

Average urge ratings are shown in Fig. 8. The planned comparisons revealed that near-misses triggered significantly greater urge than losses, *t*(56) = 1.95, *SE* = .19, *p* = .05. The planned comparison between urge ratings for wins and losses was not statistically significant, *t*(56) = −.52, *SE* = .24, *p* = .60. Additionally, the planned comparison between urge ratings for wins and near-misses was also not statistically significant, *t*(56) = −1.11, SE = .22, *p* = .27.

## Discussion

In the present study, participants experienced three types of outcomes in Candy Crush during 30 min of playing a real version of the game: Wins (when they levelled up), full losses (when they failed to level up) and near-misses (when they came close to levelling up). Based on previous research, we expected wins to be highly arousing, highly rewarding, and highly motivating.

At the most general level, our heart rate findings show that compared to the baseline epoch, playing Candy Crush is an exciting, arousing experience. Heart rate for game play was elevated compared to baseline heart rate. Importantly, such increases in arousal were likely due to game excitement as opposed to differences in the movements required in the game compared to the baseline condition. As a baseline, we specifically chose a non-exciting (rotor pursuit) task which nonetheless required finger movements comparable to those required in the game. This greater HR reactivity during gameplay has been consistently observed in previous research even when (as in this study) researchers controlled for metabolic demands due to movement (Turner et al. 1983; Carroll et al. 1984).

Our analysis of changes in skin conductance levels provided some, albeit weaker, converging evidence for this relationship. Baseline periods were associated with the largest decreases in SCLs, converging with the lowest heart rates. We note however, that for SCL changes, the baseline was only different from wins, not from losses or near-misses. It is quite possible that SCL changes may have been contaminated by the periodic swiping movements in the game. If so, the changes in SCL slopes over time may depend on how many moves were made in the last 30 s and the delays between swipes. As such we placed greater import on the heart rate measures which were not influenced by play movements.

As predicted, near-miss outcomes in Candy Crush produced significantly greater elevations in heart rate compared to regular losses. The subjective arousal ratings converge to show that just failing to level up in these games is significantly more arousing than not coming close to winning. It is reasonable to assume that as the number of available moves declines, players become more aroused in anticipation that they can attain a win. Specifically, the player begins to strive to make the correct moves with the expectation that a win is close at hand. Such mentations serve to increase heart rate and subjective arousal. When, however, they run out of moves just prior to levelling up players become frustrated (as evidenced by their high frustration ratings)—significantly more frustrated than for regular losses. Such frustration is nonetheless highly motivating, as players report greater urge to play following near-misses than following regular losses. Although near-misses are objectively equivalent to regular losses in that neither outcome results in goal attainment, gambling research suggests that near-misses trigger the urge to continue play and can lead to excessive play (Clark et al. 2009; Côté et al. 2003; Billieux et al. 2012).

As predicted, wins were highly arousing both subjectively and physiologically as evidenced by notable augmentations in heart rate and subjective ratings of arousal. They also appeared to be rewarding—wins triggered longer post-reinforcement pauses than any other outcome. Yet, despite their rewarding properties, urge following wins did not differ statistically from urge ratings following losses or near-misses. Since Candy Crush wins are periodic and unpredictable (likely occurring in a random ratio schedule similar to slot machines), it was expected that wins would be a powerful reinforcement of behaviour in this context (Ferster and Skinner 1957; Haw 2008). The absence of significantly greater urge ratings for wins compared to losses and near-misses warrants further investigation in order to reliably understand the reinforcing nature of wins in Candy Crush. If players are less inclined to continue play following wins, it can be speculated that winning may actually be a natural stopping point for these players as the incentive to continue may temporarily dwindle with goal achievement (e.g. completing a level) (Berridge 2004).

The findings concerning how near-misses trigger increases in the urge to continue play are particularly intriguing. They suggest that anticipatory arousal can be a primary motivator of future behaviour without the necessity of monetary reward. This finding supports the contention that near-misses can impact motivation regardless of the nature of the reward (Anderson and Brown 1984; Brown 1986). The anticipatory arousal prior to the near-miss in Candy Crush and the frustration that follows appears to be potent enough to invigorate further play even in a game where there is no possibility of monetary gain. Thus, Candy Crush appears to be intrinsically motivating to players, and near-misses invigorate this motivation to the similar extent of wins as demonstrated in the gambling literature (Clark et al. 2012; Côté et al. 2003; Kassinove and Schare 2001; Billieux et al. 2012).

In sum, we show that near-misses have profound effects on arousal, frustration and urge even in games where there is no possibility of monetary reward. These findings may have implications for more complex videogames as discussed by Karlsen (2011). For example, in the realm of Massive Multiplayer Online Role Playing Games (MMORPG), the arousal, frustration and urge-to-continue triggered by near-misses (just failing to achieve an objective) may impact decision-making in terms of when to quit a given game session.

One limitation of this study was our inability to show near-miss induced increases in arousal using both heart rate and skin conductance levels. Although the predicted effects were shown in heart rate, we failed to find converging evidence from our skin conductance measures. Because we used a real version of Candy Crush we could not manipulate (and counterbalance) the order in which outcomes occurred. Additionally, the fact that we had participants play levels slightly below their skill level and on a tablet rather than on a participants preferred device, may have disrupted the naturalism of game play. Thus, future research could consider having players play games directly from their device at their current level of success to maximize the potential of affective responses to outcome events. Moreover, since the effect of Candy Crush near-misses on urge has been observed, future research can investigate the behavioural consequence of such urge in terms of persistence in play.

Another limitation of this study concerns the post-reinforcement pause lengths following winning outcomes. In Candy Crush, following a winning outcome the players experience a series of eye-catching animations and exciting sounds. Unlike in slots games where players can, with the press of a button, immediately advance to the next game, Candy Crush players must wait until the cessation of these animations before playing a new game in naturalistic play. Thus, had we used the temporal duration between the outcome being revealed and the actual initiation of the next game as a measure of PRPs, these PRPs would be artificially inflated following wins by the presence of the uninterruptable animations. To circumvent this problem, we had players press an external button when they were ready to answer the subjective questions pertaining to that button. Thus, theoretically they could press button this at any time following outcome delivery (either immediately, or following a delay of variable length). Although a substantial portion of our sample initiated a button press during the playing of the animations, some players waited until the end of the animations. Thus it is difficult to get a precise estimate of the length of the true post-reinforcement pause for all participants. To get such a precise estimate, one would have to remove the animations that are played following wins—an empirical move that would dramatically reduce ecological validity.

In our version of Candy Crush, we did not subject players to “lock out” periods. In normal game play, players are only given a few chances to level up before being “locked-out” of the game for a set period of time (i.e., 30 min). Yet, to circumvent this waiting period, players are given the opportunity to make a purchase (termed micro-transaction) to resume play immediately. Although we did not use “lock out” periods our findings nonetheless may have implications toward this micro-purchasing behaviour. Specifically, as players get “locked out” following a certain number of failures, the combination of frustration and urge to continue play following a near-miss may lead players to actually pay to continue their play. Recent research has suggested that micro-transactions made in casual games, especially in those that feature gambling relevant themes, are a risk factor to migration to online gambling—even in players who have not engaged in the activity before (Kim et al. 2015). This link is especially concerning considering that players who engage in regular social games like Candy Crush may potentially play gambling relevant social games as well (King et al. 2015). Such purchasing behaviour made in these games may as such have broader, more nefarious implications. Naturally, more investigation is necessary to actually demonstrate that near-misses preferentially influence one’s decision to make micro-transactions in these types of games.

## Conclusion

In conclusion, the present study demonstrated that Candy Crush near-misses appear to have similar psychological and physiological impacts on Candy Crush players as slot-machine near-misses have on gamblers. Specifically, Candy Crush near-misses, just like their gambling-game counterparts, are physiologically arousing, and frustrating, yet motivate the urge to play.
